# TK2268 encodes the major aminotransferase involved in the conversion from oxaloacetic acid to aspartic acid in *Thermococcus kodakarensis*

**DOI:** 10.1128/aem.02017-24

**Published:** 2025-02-24

**Authors:** Yu Su, Yuta Michimori, Yuto Fukuyama, Shigeru Shimamura, Takuro Nunoura, Haruyuki Atomi

**Affiliations:** 1 Department of Synthetic Chemistry and Biological Chemistry, Graduate School of Engineering, Kyoto University 427318 , Kyoto, Japan; 2 Research Center for Bioscience and Nanoscience (CeBN), Japan Agency for Marine-Earth Science and Technology (JAMSTEC) 13570 , Yokosuka, Kanagawa, Japan; 3 Institute for Extra-Cutting-Edge Science and Technology Avant-Garde Research (X-star), Japan Agency for Marine-Earth Science and Technology (JAMSTEC) 13570 , Yokosuka, Kanagawa, Japan; 4 Integrated Research Center for Carbon Negative Science, Kyoto University 12918 , Kyoto, Japan; University of Nebraska-Lincoln, Lincoln, Nebraska, USA

**Keywords:** archaea, metabolism, aminotransferase, aspartate biosynthesis

## Abstract

Amino acid metabolism in archaea in many cases differs from those reported in bacteria and eukaryotes. The hyperthermophilic archaeon *Thermococcus kodakarensis* possesses an incomplete tricarboxylic cycle, and the biosynthesis pathway of aspartate is unknown. Here, four Class I aminotransferases in *T. kodakarensis* encoded by TK0186, TK0548, TK1094, and TK2268 were examined to identify the enzyme(s) responsible for the conversion of oxaloacetate to aspartate. Among the four proteins, the TK2268 protein (TK2268p) was the only protein to recognize oxaloacetate as the amino acceptor. With oxaloacetate, TK2268p only recognized glutamate as the amino donor. The protein also catalyzed the reverse reaction, the transamination between aspartate and 2-oxoglutarate. Substrate inhibition was observed in the presence of high concentrations of oxaloacetate or 2-oxoglutarate. Aminotransferase activity between oxaloacetate and glutamate was observed in cell extracts of the *T. kodakarensis* host strain KU216. Among the individual gene disruption strains of the four aminotransferases, a significant decrease in activity was only observed in the ΔTK2268 strain. *T. kodakarensis* KU216 does not display growth in synthetic amino acid medium when aspartate/asparagine are absent. Growth was restored upon the addition of both oxaloacetate and glutamate. Although this restoration in growth was maintained in ΔTK0186, ΔTK0548, and ΔTK1094, growth was not observed in the ΔTK2268 strain. Our results suggest that TK2268p is the predominant aminotransferase responsible for the conversion of oxaloacetate to aspartate. The growth experiments and tracer-based metabolomics using ^13^C_3_-pyruvate indicated that pyruvate is a precursor of aspartate and that this conversion is dependent on TK2268p.

**IMPORTANCE:**

Based on genome sequence, the hyperthermophilic archaeon *Thermococcus kodakarensis* possesses an incomplete tricarboxylic cycle, raising questions on how this organism carries out the biosynthesis of aspartate and glutamate. The results of this study clarify two main points related to aspartate biosynthesis. We show that aspartate can be produced from oxaloacetate and identify TK2268p as the aminotransferase responsible for this reaction. The other point demonstrated in this study is that pyruvate can act as the precursor for oxaloacetate synthesis. Together with previous results, we can propose some of the roles of the individual aminotransferases in *T. kodakarensis*. TK0548p and TK0186p are involved in amino acid catabolism, with the latter along with TK1094p involved in the conversion of glyoxylate to glycine. TK2268p is responsible for the biosynthesis of aspartate from oxaloacetate.

## INTRODUCTION

The biosynthesis and degradation of amino acids in archaea in many cases rely on enzymes and pathways that differ to those from eukaryotes and bacteria. For example, the hyperthermophilic archaeon *Aeropyrum pernix* synthesizes cysteine from 3-phosphoglycerate through 3-phosphohydroxypyruvate and *O*-phosphoserine (1). In the hyperthermophilic archaeon *Thermococcus kodakarensis* (previously reported as *Thermococcus kodakaraensis*), cysteine biosynthesis involves an ADP-dependent serine kinase that directly generates *O*-phosphoserine from serine (2). Notably, lysine biosynthesis via α-aminoadipate in *T. kodakarensis* involves a novel amino group carrier protein called LysW (3). Arginine degradation in this organism involves arginine synthetase, an enzyme that catalyzes the conversion of arginine to citrulline coupled with the generation of ATP (4). In many cases, these studies initiated by focusing on the absence of genes homologous to conventional genes described in bacteria and eukaryotes. On the other hand, studies focusing on the presence of multiple homologous genes on the genome, as described below, have also been reported.

*T. kodakarensis* is a sulfur-reducing heterotrophic archaeon that utilizes peptides and amino acids as carbon and energy sources (5, 6). The complete genome sequence reveals the presence of multiple genes with similar annotations on the genome (7). Studies on five copies of genes annotated as acyl-CoA synthetases revealed that all exhibited distinct substrate specificities (8–13), providing insight into the mechanisms of amino acid catabolism in members of Thermococcales. A recent study noted the fact that, based on primary structure, homologs of the Class I aminotransferases in Thermococcales can be classified into eight groups (G1 to G8) (14). The *T. kodakarensis* genome harbors members of seven of these groups. Among the seven genes, two are positioned within specific amino acid biosynthesis operons (TK0250, *his* operon; TK0260, *phe*/*tyr* operon), suggesting that the genes function in the biosynthesis of the respective amino acids. As TK0864 is located in the vicinity of multiple genes involved in cobalamin biosynthesis, we have been focusing on the metabolic roles of the remaining four genes (TK0186, TK0548, TK1094, and TK2268) (14, 15).

**Fig 1:**
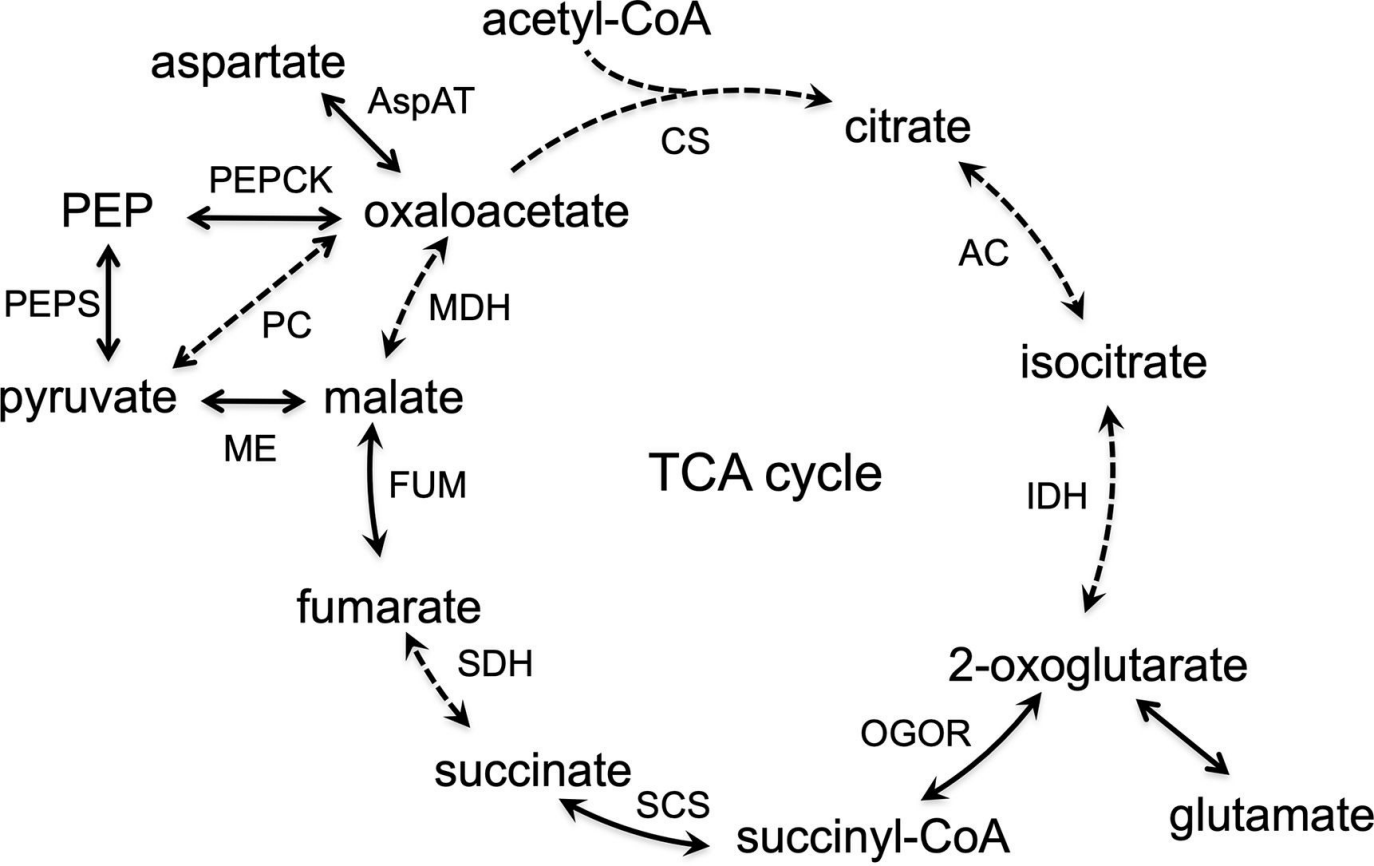
Annotation predicts an incomplete TCA cycle in *T. kodakarensis*. Genes predicted to encode enzymes of the TCA cycle were searched for on the *T. kodakarensis* genome. Solid arrows indicate the presence of homologs on the genome, while the dotted arrows indicate their absence. PEP, phosphoenolpyruvate; CS, citrate synthase; AC, aconitase; IDH, isocitrate dehydrogenase; OGOR, 2-oxoglutarate:ferredoxin oxidoreductase; SCS, ADP-forming succinyl-CoA synthetase; SDH, succinate dehydrogenase; FUM, fumarase; MDH, malate dehydrogenase; ME, malic enzyme; PEPCK, phosphoenolpyruvate carboxykinase; PC, pyruvate carboxylase; PEPS, phosphoenolpyruvate synthase; AspAT, aspartate aminotransferase.

Judging from the genome sequence, *T. kodakarensis* does not harbor a complete tricarboxylic (TCA) cycle (Fig. 1). There are no homologs for genes encoding citrate synthase, succinate dehydrogenase, and malate dehydrogenase. *T. kodakarensis* does possess genes encoding 2-oxoglutarate:ferredoxin oxidoreductase (16), ADP-forming succinyl-CoA synthetase (11), and fumarase. The former two genes have been genetically and/or biochemically established to exhibit these activities. However, the portion of the TCA cycle involving C4 acids, fumarate, malate, and oxaloacetate (OAA), is still unclear. On the other hand, enzymes in *T. kodakarensis* that might be involved in the interconversion between C3 (pyruvate, phosphoenolpyruvate [PEP]) and C4 (OAA, malate) compounds have been studied. The presence of malic enzyme (17) and phosphoenolpyruvate carboxykinase (18), which are important enzymes in C3-C4 metabolism, have been confirmed in *T. kodakarensis*, suggesting that, in principle, OAA can be generated in this organism.

OAA serves as the immediate precursor for aspartate (Asp), and the last step in the Asp biosynthesis pathway is the conversion of OAA to Asp. Both Asp aminotransferase and Asp dehydrogenase are capable of catalyzing this conversion. However, examination of cell-free extracts revealed the absence of Asp dehydrogenase in *T. kodakarensis* (16), suggesting that Asp aminotransferase is responsible for generating Asp from OAA in this organism. Asp aminotransferase belongs to Class I aminotransferases (19). In Thermococcales, a member of G4 Class I aminotransferases from *Pyrococcus furiosus* (encoded by PF1702) has been shown to exhibit Asp aminotransferase activity (20). We have shown that in *T. kodakarensis*, the TK2268 protein (TK2268p) displays Asp aminotransferase activity using pyruvate as an amino acceptor. On the other hand, the TK0548 protein (TK0548p) functions as an aromatic amino acid aminotransferase, exhibiting high activity toward Trp, Tyr, Phe, and His. Furthermore, cell-free extract experiments showed that TK0548p is responsible for nearly all His aminotransferase activities (14). The TK0186 protein (TK0186p) displays high activity toward a variety of amino acids, including Ala, Glu, Phe, Ile, Lys, Leu, Met, and Val, when glyoxylate was used as an amino acceptor (15). The TK1094 protein (TK1094p) displays high activity toward Ala and Glu with glyoxylate as the amino acceptor (15, 21). Among these four aminotransferases, TK2268p is the only aminotransferase that displays activity toward Asp (14). Nevertheless, the capacity to utilize OAA as an amino acceptor for generating Asp is still unclear. To obtain insight into Asp biosynthesis in *T. kodakarensis*, here, we examined whether these four aminotransferases can utilize OAA as an amino acceptor to produce Asp, and if so, which specific aminotransferase is responsible for this conversion.

## RESULTS

### Examination of four aminotransferases for their ability to utilize OAA as the amino acceptor

**Fig 2:**
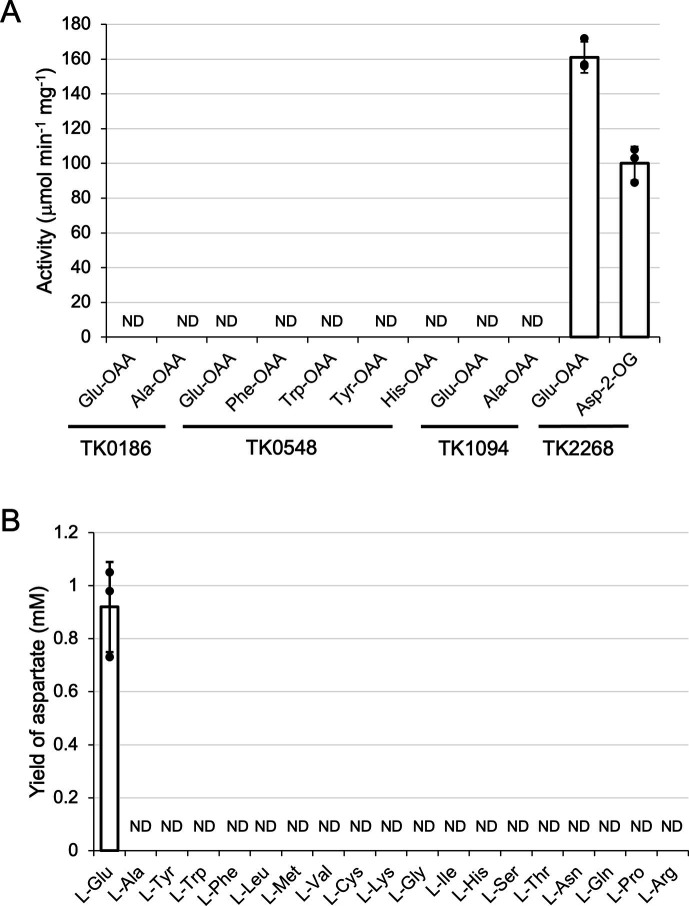
Aminotransferase activity of four aminotransferases with OAA as the amino acceptor. (**A**) Aminotransferase activity of recombinant TK0186p, TK0548p, TK1094p, and TK2268p with OAA as the amino acceptor. The amino acids that the individual proteins displayed high activity with in the presence of other amino acceptors (14, 15) and Glu were used as the amino donor. In the case of TK2268p, 2-oxoglutarate was also used as the amino acceptor. (**B**) Examination of the amino donors recognized by recombinant TK2268p with OAA as the amino acceptor. In all reactions, concentrations of both amino acceptor and donor were 10 mM. Reactions were carried out at 80°C for 10 min and the generation of Asp was examined. ND: concentrations lower than 0.02 mM were defined as not detected. The results are the means of three technically independent assays, and error bars indicate standard deviation.

Previously, the aminotransferase activities of TK0186p, TK0548p, TK1094p, and TK2268p were studied using amino acceptors other than OAA (14, 15). This was in part due to the fact that we experienced difficulties in separation of Asp and Glu in HPLC analyses. By improving the composition of the mobile phase, we were able to clearly detect either of the two amino acids even in the presence of high concentrations of the other. To examine which of the four aminotransferases could catalyze the transamination reaction using OAA as the amino donor, we incubated each protein using Glu and OAA as substrates (Fig. 2A). We observed the generation of Asp only with TK2268p. As the lack of Asp-generating activity of TK0186p, TK0548p, and TK1094p may be due to the fact that these proteins do not recognize Glu as the amino donor, we also examined the activity with OAA and amino donors that have been shown to be preferred by these proteins: Ala for TK0186p, Phe, Trp, Tyr, and His for TK0548p and Ala for TK1094p (14, 15). As a result, none of the other proteins displayed activity with OAA as the amino acceptor. To determine the amino acids that can be recognized by TK2268p when OAA was used as the amino acceptor, various amino acids were used as amino donor and the generation of Asp was examined. As indicated in Fig. 2B, generation of Asp was clearly observed only when Glu was used as the amino donor. Intriguingly, Ala as an amino donor did not lead to significant levels of Asp generation although the protein displayed activity for the reverse reaction, converting Asp and pyruvate to oxaloacetate and Ala (14). The results suggested that TK2268p specifically utilizes Glu as the amino donor when OAA is used as the amino acceptor. TK2268p also catalyzed the reverse reaction (transamination between Asp and 2-oxoglutarate) with high aminotransferase activity (Fig. 2A). These results suggested that among these four Class I aminotransferases, TK2268p is the only enzyme that displays high aminotransferase activity resulting in the generation of Asp.

### Kinetic analysis

**Fig 3:**
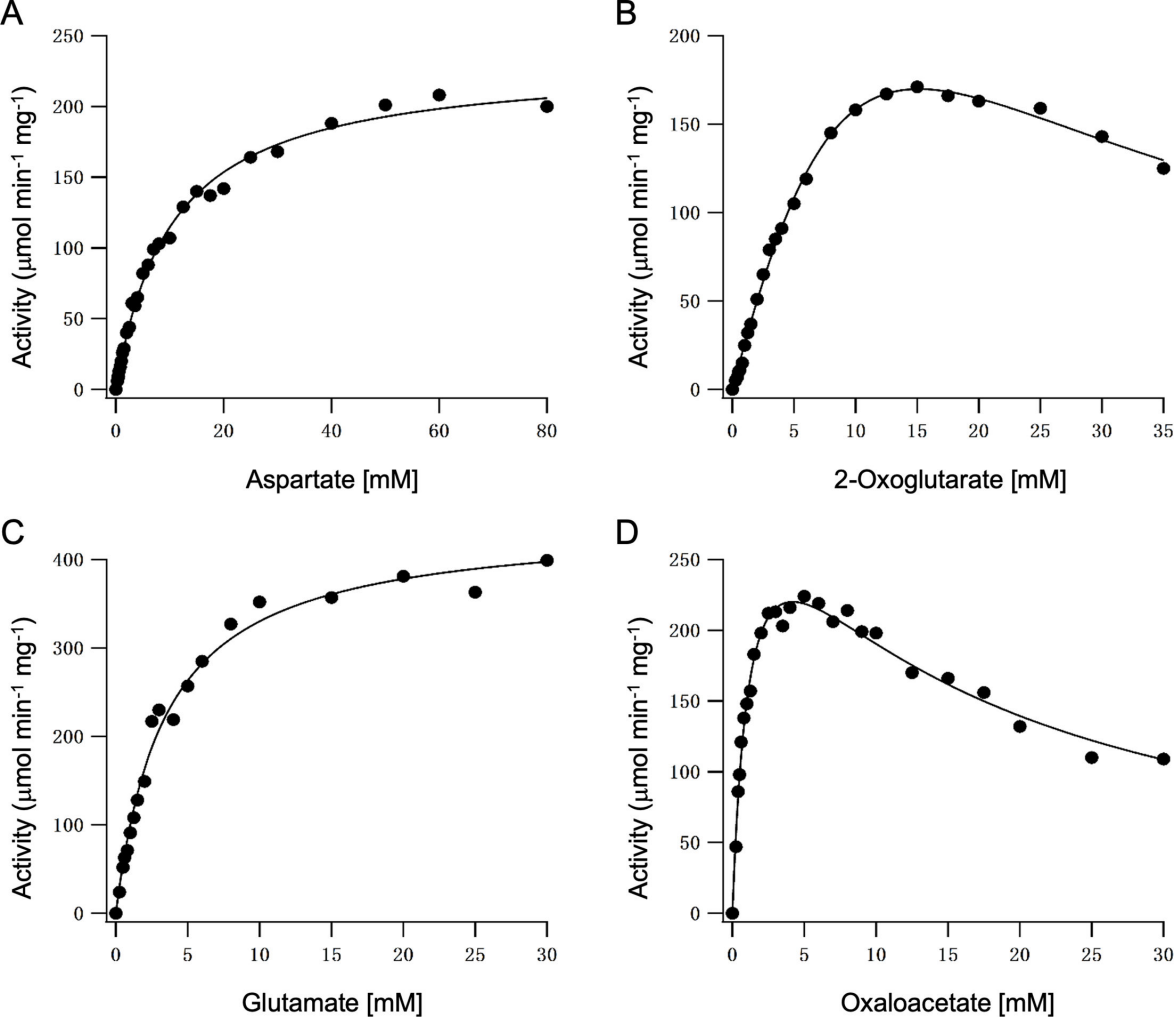
Kinetic characterization of the TK2268p reaction. (**A**) Initial velocity with various concentrations of Asp and 10 mM 2-oxoglutarate. (**B**) Initial velocity with various concentrations of 2-oxoglutarate and 10 mM Asp. (**C**) Initial velocity with various concentrations of Glu and 10 mM OAA. (**D**) Initial velocity with various concentrations of OAA and 4 mM Glu.

Kinetic analysis of the Asp aminotransferase reaction catalyzed by TK2268p was performed. TK2268p displayed typical Michaelis-Menten kinetics toward the amino donors Glu and Asp (Fig. 3). However, substrate inhibition was clearly observed toward the amino acceptors OAA and 2-oxoglutarate. Our data fit well to a typical substrate inhibition model, expressed as *v* = *V*_max_[S]/(*K*_s1_ + [S] + [S]^2^/*K*_s2_), where *v* is reaction velocity, *V*_max_ is maximum velocity, [S] is substrate concentration, *K*_s1_ is the dissociation constant between enzyme and the first substrate, and *K*_s2_ is the dissociation constant between the enzyme-substrate complex and the second, inhibitory substrate. As shown in Table 1, the *K*_s1_ for OAA is much lower than the *K*_m_ for Asp and *k*_cat_/*K*_s1_ for OAA is more than 10-fold higher than the *k*_cat_/*K*_m_ for Asp and the *k*_cat_/*K*_m_ for Glu is more than 4-fold of the *k*_cat_/*K*_s1_ for 2-oxoglutarate. The results suggest that TK2268p favors the reaction from OAA and Glu to Asp and 2-oxoglutarate and raises the possibility that the protein plays an important role in Asp biosynthesis in *T. kodakarensis*.

**TABLE 1:** Kinetic parameters of the aminotransferase reaction catalyzed by TK2268p*^a^*

Substrate		*V*_max_ (μmol min^−1^ mg^−1^)	*K*_m_ (mM)	*k*_cat_ (s^−1^)	*k*_cat_/*K*_m_ (mM^−1^ s^−1^)
Amino donor	Amino acceptor				
*Aspartate*	2-Oxoglutarate^*b*^	232 ± 4	10.3 ± 0.5	175 ± 4	17.0
*Glutamate*	OAA^*b*^	443 ± 11	3.4 ± 0.3	333 ± 12	97.8
Asp*^b^*	*2-Oxoglutarate*	1,040 ± 230	38.8±9.6^*d*^	781 ± 244	20.1^*f*^
			5.9 ± 1.6^*e*^		
Glutamate^*c*^	*Oxaloacetate*	360 ± 15	1.3±0.1^*d*^	270 ± 16	208^*f*^
			13.2 ± 1.2^*e*^		

^*a*^ The kinetic parameters are those for the substrates indicated in italic.

^*b*^ Concentration fixed at 10 mM.

^*c*^ Concentration fixed at 4 mM.

^*d*^ *K*_s1_.

^*e*^ *K*_s2_.

^*f*^ *k*_cat/_ *K*_s1._

### Asp-generating aminotransferase activities in cell-free extracts

**Fig 4:**
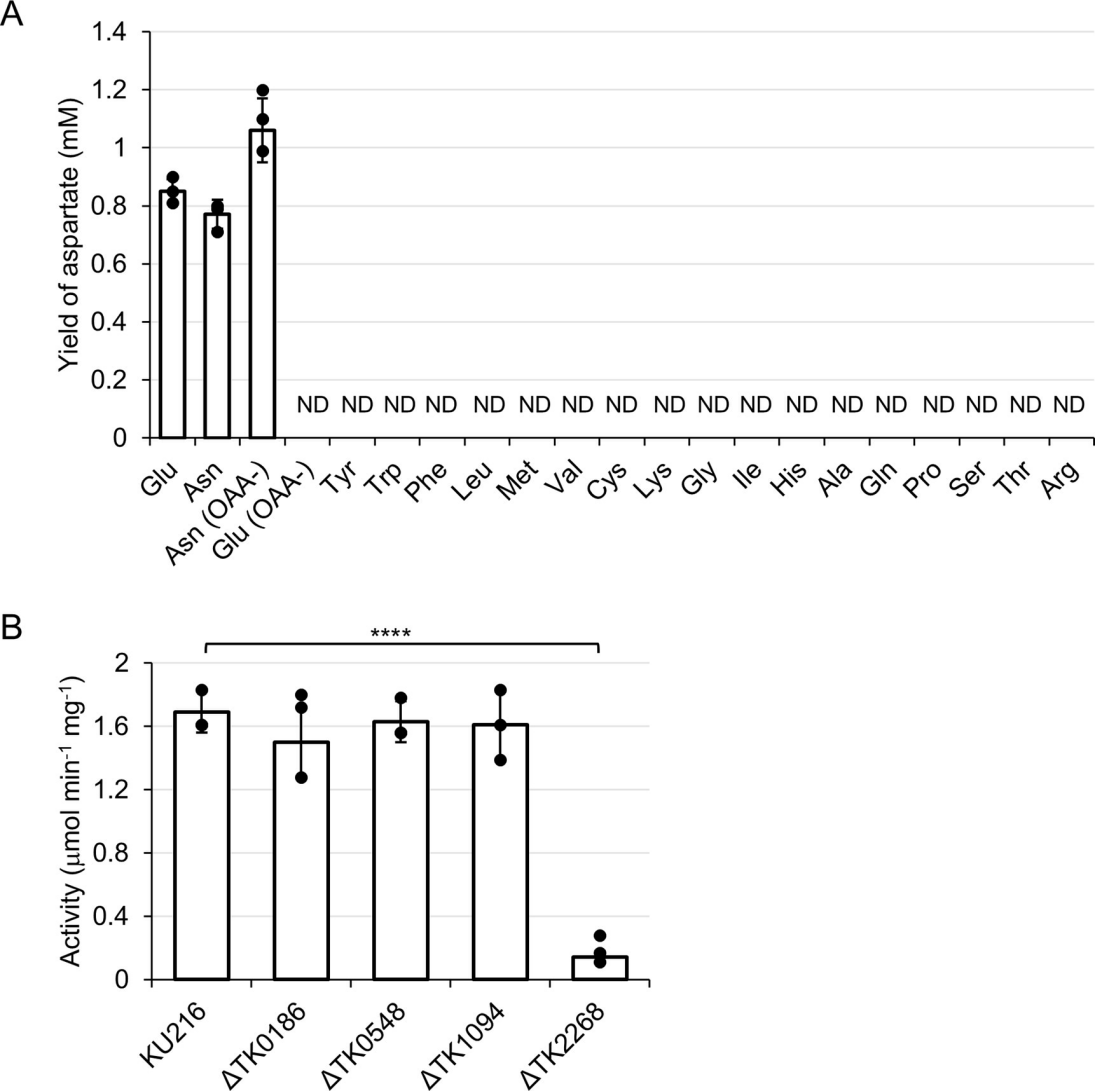
Asp-generating aminotransferase activity in cell-free extracts of *T. kodakarensis*. (**A**) Asp-generating activity in the cell-free extract of the host strain KU216. The reaction mixture was incubated at 80°C for 15 min with the indicated amino acids or their sodium salts and OAA, and the generation of Asp was examined. Reaction mixtures with sodium Asn or Glu without sodium OAA were used as a control. Amino acids and OAA were added at a concentration of 10 mM. (**B**) Asp-generating aminotransferase activity in cell-free extracts of different *T. kodakarensis* strains. Cell extracts were incubated with sodium OAA and sodium Glu at 80°C for various periods of time. Activity was calculated based on the generation of Asp. The final concentrations of Glu and OAA were 10 mM. 0.0001 < *P* < 0.001: ****. The results are the means of three technically independent assays, and error bars indicate standard deviation.

To evaluate the Asp-generating aminotransferase activity *in vivo*, the activity in the cell-free extract of *T. kodakarensis* KU216 grown in ASW-YT-m1 medium supplemented with 5 g L^−1^ sodium pyruvate was examined. *T. kodakarensis* KU216 is a strain deleted of the orotidine-5′-monophosphate decarboxylase gene (*pyrF*) and displays uracil auxotrophy in synthetic medium (22, 23). The results with 19 amino acids (excluding Asp) as the amino donor and OAA as the amino acceptor (Fig. 4A) indicated that only Glu was recognized as the amino donor when OAA was used as the amino acceptor. Asp generation was observed when asparagine (Asn) was used as the amino donor. However, the generation of Asp from Asn was also observed when OAA was removed from the reaction mixture (Fig. 4A), suggesting that the generation of Asp in this case is due to the presence of asparaginase in *T. kodakarensis*, which has been biochemically examined in a previous study. We next examined the Asp-generating aminotransferase activity in the cell extracts from the individual gene disruption strains. These strains were constructed using *T. kodakarensis* KU216 as the parent strain. We found that cell-free extracts of ΔTK0186, ΔTK0548, and ΔTK1094 displayed Asp-generating aminotransferase activities comparable to that of the host strain. In contrast, a significant decrease (approximately 90%) in Asp-generating activity was observed in the cell-free extract οf ΔTK2268 (Fig. 4B), suggesting that TK2268p is the predominant aminotransferase responsible for the generation of Asp from OAA *in vivo*.

### Growth experiments of the four aminotransferase gene disruption strains

The contributions of the TK0548 and TK2268 genes to amino acid catabolism in *T. kodakarensis* have been previously reported (14). In this study, the contributions of the TK0186 and TK1094 genes were studied by cultivating the ΔTK0186 and ΔTK1094 strains in the synthetic medium ASW-AA-m1-S^0^(Ura^+^). Cells were initially grown in nutrient-rich media, followed by cultivation in ASW-AA-m1-S^0^(Ura^+^). After growth, cells were then inoculated again to ASW-AA-m1-S^0^(Ura^+^) and the results are shown in Fig. S1. In previous studies (14), growth in ASW-AA-m1-S^0^(Ura^+^) was examined with cells inoculated directly from nutrient-rich media, so we also examined the growth of ΔTK0548 and ΔTK2268 under the conditions applied here. The results showed that the disruption of three genes, TK0186, TK0548, and TK2268, resulted in a slight impairment of growth in ASW-AA-m1-S^0^(Ura^+^), suggesting that the genes are involved in the utilization of amino acids in *T. kodakarensis* (Fig. S1). On the contrary, we did not observe effects on growth upon disruption of TK1094. TK1094p displayed activity with a high preference toward Ala and Glu (15). There is a possibility that the activity of TK1094p can be compensated by TK0186p under these conditions, as the latter protein has been shown to also display significant activity toward Ala and Glu, among other amino donors (15).

### Involvement of TK2268 in Asp biosynthesis

To examine whether *T. kodakarensis* can synthesize Asp/Asn from the other 18 amino acids, *T. kodakarensis* KU216 was cultivated in synthetic medium free of aspartate and asparagine (Asp/Asn), ASW-AA(D^−^N^−^)-m1-S^0^(Ura^+^). In comparison to the robust growth observed in a synthetic medium containing all 20 amino acids (ASW-AA-m1-S^0^(Ura^+^)), *T. kodakarensis* KU216 did not exhibit growth in medium without Asp/Asn after incubation at 85°C for 4 d (results through 3 d are shown in Fig. S2A). The lack of growth when Asp/Asn are not available suggested that Asp is essential for the growth of *T. kodakarensis* in this medium, and it cannot be sufficiently synthesized from the other 18 amino acids at the applied concentrations.

As our biochemical analyses indicated that TK2268p is specific for the conversion between OAA/Glu and Asp/2-oxoglutarate, we examined whether OAA could complement the Asp/Asn auxotrophy of *T. kodakarensis* KU216. KU216 was cultivated in ASW-AA(D^−^N^−^)-m1-S^0^(Ura^+^)-OAA, a synthetic medium supplemented with 5 g L^−1^ sodium OAA. Growth was not observed in this medium. However, when we further supplemented the medium with 2 g L^−1^ Glu as an amino donor (ASW-AA(D^−^N^−^)-m1-S^0^(Ura^+^)-OAA-Glu medium), we observed growth of KU216 (Fig. S2B). We confirmed that KU216 does not display growth in medium supplemented with Glu alone (ASW-AA(D^−^N^−^)-m1-S^0^(Ura^+^)-Glu), ruling out the possibility that Asp is synthesized from Glu independent of an aminotransferase reaction. These results suggest that *T. kodakarensis* can synthesize Asp from OAA and Glu, most likely through the function of TK2268p.

**Fig 5:**
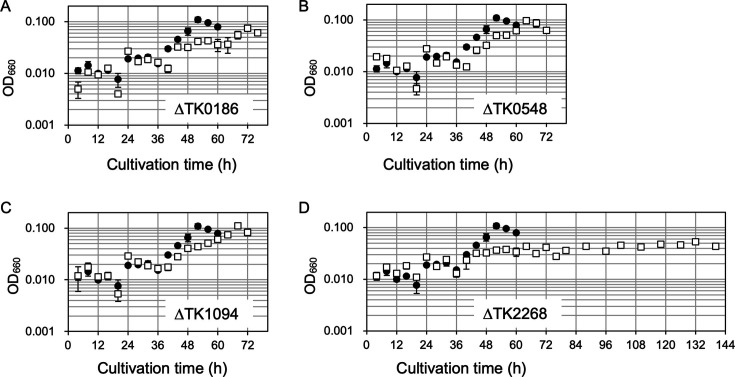
Growth of *T. kodakarensis* KU216 and aminotransferase gene disruption strains in synthetic medium ASW-AA(D^−^N^−^)-m1-S^0^(Ura^+^) with OAA and Glu. A, B, C, and D display the effects of OAA and Glu on the growths of ΔTK0186, ΔTK0548, ΔTK1094, and ΔTK2268 strains, respectively. Growth of KU216 is indicated with closed circles, and those of the disruption strains are indicated with open squares. Error bars indicate the standard deviation values of three biologically independent culture experiments.

**Fig 6:**
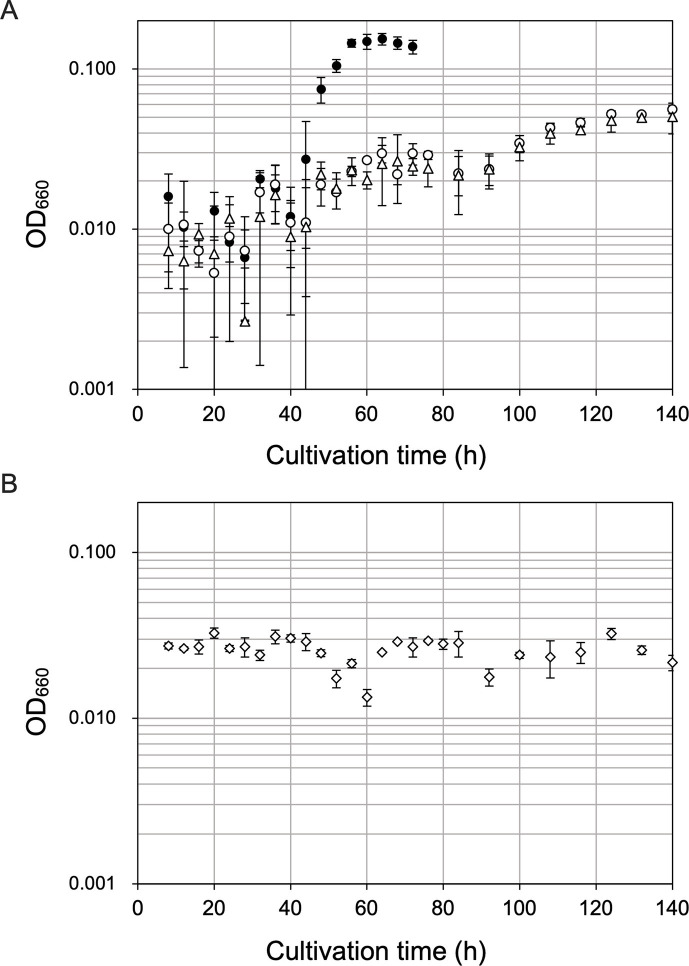
Growth properties of *T. kodakarensis* KU216 and ΔTK2268 in various synthetic media. (**A**) *T. kodakarensis* KU216 (closed circles) and ΔTK2268 (open circles) cultivated in ASW-AA(D^−^N^−^)-m1-S^0^(Ura^+^) supplemented with sodium pyruvate and Glu. Measurements of a culture without cell inoculation are shown with open triangles. (**B**) *T. kodakarensis* KU216 cultivated in ASW-AA(D^−^N^−^)-m1-S^0^(Ura^+^) supplemented with sodium malate and Glu (opened diamonds). Error bars indicate the standard deviation values of three biologically independent culture experiments.

Το add further support, the ΔTK0186, ΔTK0548, ΔTK1094, and ΔTK2268 strains were cultivated in the synthetic medium ASW-AA(D^−^N^−^)-m1-S^0^(Ura^+^)-OAA-Glu. Although a delay in growth was observed, the ΔTK0548 and ΔTK1094 strains both displayed growth comparable with that of the host strain KU216 (Fig. 5B and C). In contrast, the ΔTK0186 strain displayed a more serious growth impairment and the maximum cell yield decreased by ~30% (Fig. 5A). As growth in this medium reflects the sum of both the ability to utilize amino acids for carbon/energy and the ability to synthesize Asp/Asn, the impairments in growth observed in these three strains may be due to the effects of gene disruption on amino acid utilization. In contrast to the growth observed in the above three strains, the ΔTK2268 strain did not display any growth in this medium for at least 5 d (Fig. 5D). The gradual increase in optical density (OD) is the result of browning of the medium, as this is also observed in medium without cell inoculation (see Fig. 6A). The results suggest that TK2268 is necessary for Asp biosynthesis from OAA in *T. kodakarensis*, at least to synthesize sufficient levels of Asp to sustain growth. Along with our biochemical analyses, the results obtained here strongly suggest that TK2268 encodes the major aminotransferase responsible for Asp biosynthesis from OAA in *T. kodakarensis*.

### Source of OAA in *T. kodakarensis*

In many bacteria and eukaryotes that harbor a TCA cycle, OAA is metabolically linked with malate via malate dehydrogenase and citrate via citrate synthase. OAA can also be formed from pyruvate (pyruvate carboxylase), phosphoenolpyruvate (PEP) (PEP carboxylase or PEP carboxykinase), or citrate (e.g., ATP citrate lyase). However, *T. kodakarensis* does not harbor many homologs of TCA cycle enzymes, including malate dehydrogenase, citrate synthase/lyase, and succinate dehydrogenase (Fig. 1), making it difficult to predict the source of OAA. In order to evaluate the source of OAA, we replaced OAA in ASW-AA(D^−^N^−^)-m1-S^0^(Ura^+^)-OAA-Glu with malate or pyruvate (Fig. 6). We observed growth when OAA was replaced with pyruvate, but not with malate, suggesting that OAA can be supplied from pyruvate, either directly or possibly through PEP. The presence of a PEP carboxykinase in *T. kodakarensis* has been experimentally established (18), and along with a genetically examined PEP synthase that can convert pyruvate to PEP, the presence of a route from pyruvate to OAA is suggested.

If pyruvate were to be a source of OAA, the effects of TK2268 gene disruption on growth in ASW-AA(D^−^N^−^)-m1-S^0^(Ura^+^)-Pyr-Glu should be similar to that observed in ASW-AA(D^−^N^−^)-m1-S^0^(Ura^+^)-OAA-Glu. As shown in Fig. 6, although KU216 could grow in ASW-AA(D^−^N^−^)-m1-S^0^(Ura^+^)-Pyr-Glu, ΔTK2268 could not grow in this medium, suggesting that the pyruvate added to this medium is converted to Asp via OAA and not by a route independent of TK2268.

### Evaluation of the conversion of pyruvate to Asp with ^13^C tracer-based metabolomics

**Fig 7:**
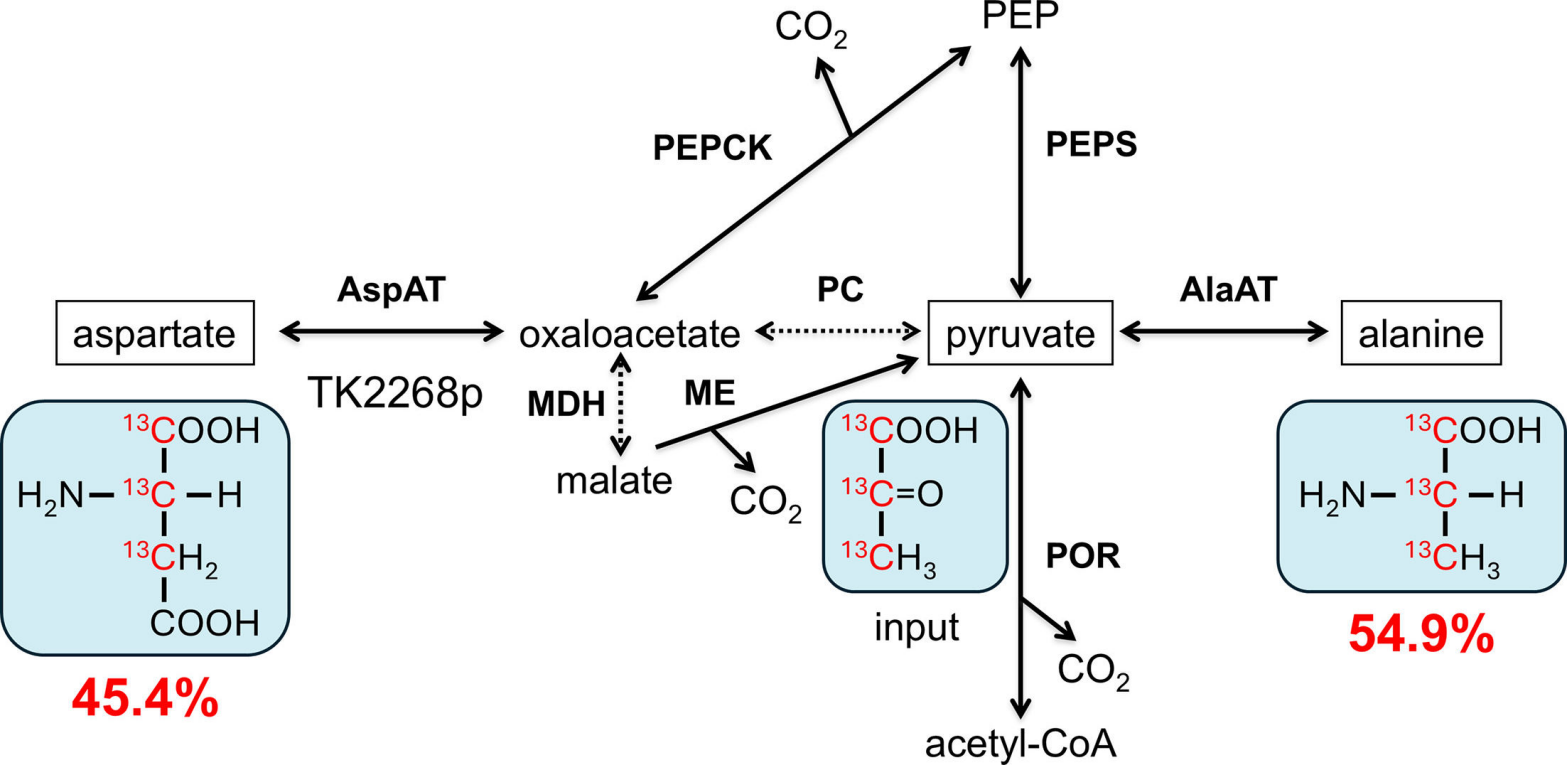
The relative abundance of ^13^C-labeled Ala and Asp derived from ^13^C_3_-pyruvate.*T. kodakarensis* cells were grown in a synthetic medium without Asp/Asn with the addition of ^13^C_3_-pyruvate (see text). Major isotopomers of Ala and Asp, along with their relative abundance, are shown. Possible metabolic routes from pyruvate to Ala and Asp are presented. Solid arrows indicate the presence of homologs on the genome, while the dotted arrows indicate their absence. Details of the isotopomer analyses are shown in Fig. S3 and S4. AlaAT, alanine aminotransferase; AspAT, aspartate aminotransferase; MDH, malate dehydrogenase; ME, malic enzyme; PC, pyruvate carboxylase; PEPCK, phosphoenolpyruvate carboxykinase; PEPS, phosphoenolpyruvate synthase; POR, pyruvate:ferredoxin oxidoreductase.

To clarify that pyruvate is actually a precursor for Asp biosynthesis in *T. kodakarensis*, the host strain *T. kodakarensis* KU216 was cultivated in the synthetic medium ASW-AA(D^−^N^−^)-m1-S^0^(Ura^+^)-Pyr-Glu supplemented with pyruvate fully labeled with ^13^C (^13^C_3_-pyruvate). After growth, proteinogenic Asp, as well as Ala, were evaluated by capillary electrophoresis (CE)-high-resolution MS. As a result, we found that 54.9% and 45.4% of Ala and Asp, respectively, were shared by a mass fraction of M+3 (Fig. 7). In addition, all the detected M+3 Asp harbored ^13^C in the C-1, 2, and 3 positions (Fig. 7). It has been well documented that Ala is generated from pyruvate in *Thermococcus* and *Pyrococcus* species (20, 24–26), and we have shown that TK1094p and TK0186p catalyze the conversion from pyruvate to Ala (15, 21). Our results also indicate that pyruvate is a precursor for Asp biosynthesis and strongly support our proposal stated above that pyruvate or PEP is carboxylated to oxaloacetate, which is then converted to Asp through the function of TK2268p. The mass fraction of M+2 observed in Ala and Asp (Fig. S3 and S4) can partially be explained by the reversible conversion of pyruvate and acetyl-CoA through the function of pyruvate:ferredoxin oxidoreductase (POR), which gradually results in the generation of [2,3-^13^C_2_] pyruvate when a nonlabeled CO_2_ molecule is used in the conversion of acetyl-CoA back to pyruvate. The other isotopomers of Ala and Asp would be derived from ^13^C_2_-pyruvate generated by unknown biosynthesis mechanisms. We also observed a mass fraction of M+4 in Asp, and this can be explained by the release of ^13^CO_2_ by POR and its re-incorporation to ^13^C_3_-pyruvate through a carboxylation reaction from pyruvate or PEP to oxaloacetate.

## DISCUSSION

The results presented here reveal the presence of a metabolic route from OAA to Asp via an aminotransferase reaction catalyzed by TK2268p. The results of growth experiments and ^13^C tracer-based metabolomic analyses suggested that other aminotransferases are not involved in this conversion. On the other hand, when comparing the activity (*k*_cat_/*K*_m_ values) of TK2268p toward other amino donors/acceptors, activity toward Glu/OAA (97.8 mM^−1^ s^−1^) was much higher than those with other substrates such as Leu/2-oxoglutarate (17.6 mM^−1^ s^−1^) (14), suggesting that the major role of the enzyme in *T. kodakarensis* is participating in the conversion between Glu/OAA and Asp/2-oxoglutarate. The only other route to generate Asp in *T. kodakarensis* is from Asn. Generation of Asp from Asn was observed in the cell extract (Fig. 4A), and in previous studies, the TK1656 protein and TK2246 protein have been demonstrated to display asparaginase activity (27, 28) .

The distribution of TK2268p homologs in 40 members of Thermococcales was examined. Closely related homologs of TK2268p were found in all 40 members with E-values less than 1 × 10^−180^ (Table S1), suggesting that all members of Thermococcales have the potential to synthesize Asp from OAA. However, how OAA is generated in *T. kodakarensis* is still unknown. The addition of pyruvate to the medium in place of OAA supported growth of *T. kodakarensis* in the absence of Asp/Asn. As this growth was not observed in ΔTK2268, it is most likely that a route exists that can generate OAA from pyruvate. This was confirmed by tracer-based metabolomics with the CE-high-resolution MS experiments using ^13^C_3_-pyruvate. On the other hand, malate could not replace OAA. This agrees well with the fact that *T. kodakarensis* does not harbor a malate dehydrogenase homolog on its genome. The potential enzyme that might be involved in the conversion from pyruvate to OAA is PEP carboxykinase (in combination with PEP synthase), which has been biochemically characterized as a GTP-dependent enzyme encoded by TK1405 (18). A malic enzyme, encoded by TK1963, has also been studied, but it is hard to imagine a scenario in which pyruvate is converted to OAA without additional energy. Further analyses will be necessary to identify the enzyme(s) responsible for the conversion from pyruvate to OAA.

**Fig 8:**
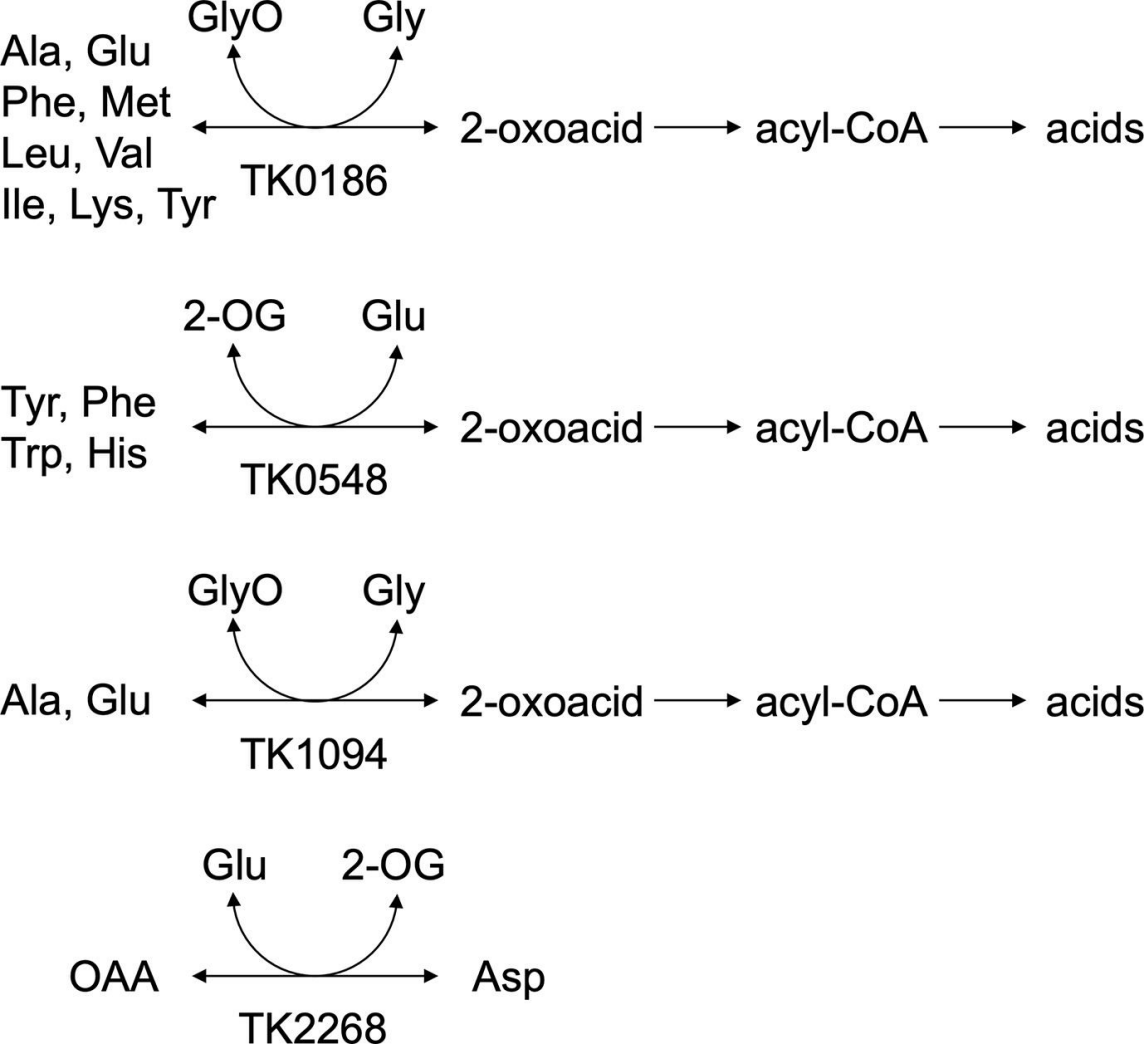
Proposed roles of the four aminotransferases encoded by TK0186, TK0548, TK1094, and TK2268 in *T. kodakarensis*. Although the aminotransferase reactions are presented as reversible reactions, the reactions catalyzed by the TK0186, TK0548, and TK1094 proteins have only been experimentally confirmed in the direction of catabolism (from left to right). The reaction catalyzed by the TK2268 protein has been confirmed in both directions. When Ala is the amino donor, the TK0186 protein can also utilize 2-oxoglutarate, 3-phenylpyruvate, 2-oxoisocaproate, 2-oxoisovalerate, and indole-3-pyruvate as the amino acceptor, while the TK1094 protein can utilize 2-oxoglutarate (15). It should be noted that enzymes responsible for the conversion of Lys following the transamination reaction are not known (12). OAA, oxaloacetate; 2-OG, 2-oxoglutarate; GlyO, glyoxylate.

Amino acid catabolism in Thermococcales is presumed to proceed via amino acid, 2-oxoacid, acyl-CoA, and finally acid (16). Amino acid dehydrogenase and/or amino acid aminotransferase catalyze the initial conversion of amino acid into their corresponding 2-oxoacids. Glu dehydrogenase has been established as the only amino acid dehydrogenase in *T. kodakarensis*, converting Glu to 2-oxoglutarate (16). This implies that the amino acid aminotransferases are responsible for the conversion of other amino acids to 2-oxoacids. Subsequently, 2-oxoacid:ferredoxin oxidoreductases and acyl-CoA synthetases catalyze the conversion of 2-oxoacid into acids. Various 2-oxoacid:ferredoxin oxidoreductases (POR, 2-ketoisovalerate:ferredoxin oxidoreductase (VOR), indolepyruvate:ferredoxin oxidoreductase (IOR), and 2-oxoglutarate:ferredoxin oxidoreductase) (29–32) in the members of Thermococcales have been biochemically or genetically studied. The substrate specificity of five NDP-forming acyl-CoA synthetases (ACS I, ACS II, ACS III, succinyl-CoA synthetase [SCS], and 2-[imidazol-4-yl]acetyl-CoA synthetase [ICS]) (8–13) in Thermococcales suggested that the amino acids that are subject to this mode of catabolism are Ala, Val, Ile, Leu, Met, Phe, Tyr, Trp, Glu/Gln, Cys, and His (12). Malonyl-CoA is not actively recognized by the acyl-CoA synthetases from *T. kodakarensis*. This suggests that Asp is not converted through this pathway and that Asp may not be a catabolic substrate. The physiological role of TK2268p is most likely related to Asp biosynthesis (Fig. 8).

Our biochemical and genetic studies here and in previous studies provide a general indication as to the metabolic roles of the four Class I aminotransferases. TK0186p recognizes a wide range of amino acids including Ala, Glu, Phe, Ile, Lys, Leu, Met, Val, and Tyr (15). An aminotransferase gene presumed necessary for the biosyntheses of Tyr, and Phe is found in their biosynthesis gene cluster (TK0260), and *T. kodakarensis* is not capable of synthesizing Ile, Leu, and Val. The amino group of Met is maintained from its precursor, Asp, and does not require an aminotransferase. The α-amino group of Lys is incorporated prior to the linking of the protecting peptide LysW (3). It can thus be presumed that the activities of TK0186p toward Phe, Ile, Lys, Leu, Met, Val, and Tyr are related to the catabolic degradation of these amino acids (Fig. 8; Table 2). The reversible Ala to pyruvate and Glu to 2-oxoglutarate can be considered central to amino acid metabolism in *T. kodakarensis*, as multiple aminotransferases can catalyze these reactions. As for the latter reaction, glutamate dehydrogenase also contributes in the conversion, providing or consuming electrons and ammonia. In summary, TK0186p is most likely involved in the catabolism of a wide range of amino acids and also contributes in the homeostasis of amino acids, nitrogen, and redox balance. TK0548p accepts His, Tyr, Trp, and Phe as the amino donor (14). Aminotransferase genes presumed as necessary for the biosyntheses of His, Tyr, and Phe are found in their respective biosynthesis gene clusters (His: TK0250, Tyr/Phe: TK0260), while Trp biosynthesis, using tryptophan synthase, does not utilize an aminotransferase reaction to introduce the α-amino group. It is thus highly likely that the physiological role of TK0548p is in the catabolic breakdown of His, Tyr, Trp, and Phe (Fig. 8; Table 2). TK1094p only accepts a narrow range of amino acids, recognizing only Ala and Glu (15). As in the case of TK0186p, TK1094p can be presumed to contribute in the homeostasis of amino acids, nitrogen, and redox balance (Fig. 8; Table 2). TK2268p is the only aminotransferase involved in Asp biosynthesis. The enzyme may also be involved in the catabolism of Leu and Tyr, as activity was observed with 2-oxoglutarate as the amino acceptor (14). The effects of gene disruption on growth in amino acid medium (Fig. S1) can thus be interpreted as follows. The decrease in cell yield in ΔTK0186 may be due to the inability of cells to convert Ile or Val. It should be noted that the conversion of aminotransferase reaction of Val provides 2-oxoisovalerate, the precursor of CoA. The growth impairment in ΔTK0548 most likely reflects the inability to catabolize His and Trp. The apparent lack of any effects on growth upon disruption of TK1094 is probably due to the fact that TK0186p (Ala/Glu) and TK2268p (Glu) can substitute for TK1094p in converting Ala and Glu. The growth defects observed for ΔTK2268 are most likely due to deficiencies in Asp biosynthesis, as catabolism of Leu and Tyr could be substituted by TK0186p (Leu, Tyr) and TK0548p (Tyr).

**TABLE 2:** Amino donors and acceptors recognized by the four aminotransferases^*a*^

Gene number	Amino donor	Amino acceptor
TK0186	Ala	2-Oxoglutarate, 3-phenylpyruvate, glyoxylate, 2-oxoisovalerate, indole-3-pyruvate, (2-oxoisocaproate)
	Ala, Glu, Phe, Leu, Met, Val, (Ile, Lys, Tyr)	Glyoxylate
TK0548	Tyr, Phe, Trp, His, (Met, Leu)	2-Oxoglutarate
	(Glu)	(Pyruvate)
TK1094	Ala, Glu, (Cys)	Glyoxylate
	Ala	2-Oxoglutarate
TK2268	Glu	Oxaloacetate
	Asp, (Cys, Leu, Ala, Met)	2-Oxoglutarate
	(Glu, Asp)	(Pyruvate)

^*a*^ Compounds presented in parentheses result in detectable, but relatively low activity.

## MATERIALS AND METHODS

### Strains and culture conditions

Chemicals were purchased from Wako Pure Chemicals (Osaka, Japan) or Nacalai Tesque (Kyoto, Japan) unless mentioned otherwise. *Thermococcus kodakarensis* KOD1 is a hyperthermophilic archaeon isolated from Kodakara Island, Kagoshima, Japan (5, 6). *T. kodakarensis* KU216 (Δ*pyrF*) (22, 23) and its derivative strains were cultivated under strictly anaerobic conditions at 85°C in nutrient-rich medium (ASW-YT-m1-S^0^ or ASW-YT-m1-pyruvate) or synthetic medium (ASW-AA-m1-S^0^). ASW-YT-m1-S^0^, ASW-YT-m1-pyruvate, and ASW-AA-m1-S^0^ are the modified versions of ASW-YT-S^0^, ASW-YT-pyruvate, and ASW-AA-S^0^ medium, respectively. ASW-YT-S^0^ was composed of 0.8× artificial seawater (ASW) (33), 5 g L^−1^ yeast extract, 5 g L^−1^ tryptone, and 2 g L^−1^ elemental sulfur. In ASW-YT-m1-S^0^, 20 µM KI, 20 µM H_3_BO_3_, 10 µM NiCl_2_·6H_2_O, and 10 µM Na_2_WO_4_ were supplemented. In ASW-YT-m1-pyruvate medium, elemental sulfur in ASW-YT-m1-S^0^ medium was replaced with 5.0 g L^−1^ sodium pyruvate. ASW-AA-S^0^ was composed of 0.8× ASW, a mixture of 20 amino acids, modified Wolfe’s trace minerals, and a mixture of vitamins (22). In ASW-AA-m1-S^0^, 20 µM KI, 20 µM H_3_BO_3_, 10 µM NiCl_2_·6H_2_O, and 10 µM Na_2_WO_4_ were supplemented, and the concentrations of arginine hydrochloride and valine were increased (from 250 mg L^−1^ to 500 mg L^−1^ and from 100 mg L^−1^ to 400 mg L^−1^, respectively); 10 µg mL^−1^ uracil was added to ASW-AA-m1-S^0^ medium to make ASW-AA-m1-S^0^(Ura^+^) medium when *pyrF* gene disruption strain was cultivated. To remove oxygen in the medium, 5% (wt/vol) Na_2_S·9H_2_O solution was added until the medium became colorless. Resazurin (0.5 mg L^−1^) was also added to all media as an oxygen indicator. *Escherichia coli* DH5α (Takara Bio, Kusatsu, Japan) and BL21-CodonPlus(DE3)-RIL strains (Agilent Technologies, Santa Clara, CA) were cultivated at 37°C in Lysogeny broth (LB) medium supplemented with ampicillin (100 mg L^−1^). *E. coli* DH5α was used for recombinant plasmid construction, and *E. coli* BL21-CodonPlus(DE3)-RIL was used for heterologous gene expression.

### Purification of the recombinant proteins

TK0548 expression plasmid was introduced into the *E. coli* BL21-CodonPlus(DE3)-RIL strain. Transformants were cultivated in LB medium (100 mg L^−1^ ampicillin and 30 mg L^−1^ chloramphenicol) at 37°C until the OD_660_ reached 0.6. Gene overexpression was induced by adding isopropyl-1-thio-*β*-D-galactopyranoside (IPTG) to a final concentration 0.1 mM followed by cultivation at 18°C for 20 h. Cells were harvested via centrifugation (5,540 × *g*, 15 min, 4°C). Cell pallet was washed with 50 mM Tris-HCl (pH 7.5) and then was resuspended. Cells were lysed by sonication, and the insoluble cell debris was separated by centrifugation (12,000 × *g*, 15 min, 4°C). The soluble cell extract was heat treated at 85°C for 15 min, and the thermolabile proteins derived from the host were removed by centrifugation (12,000 × *g*, 15 min, 4°C). The supernatant was loaded onto an anion exchange column Resource Q (GE Healthcare, Little Chalfont, Buckinghamshire, United Kingdom) equilibrated with 50 mM Tris-HCl (pH 7.5). Proteins were eluted with a linear gradient of NaCl (0–1.0 M) in 50 mM Tris-HCl (pH 7.5) at a flow rate of 2.5 mL min^−1^. Fractions that contain TK0548 protein were collected and concentrated with an Amicon Ultra centrifugal filter unit (MWCO 10 K) (EMD Millipore, Billerica, MA). The resulting protein solution was applied to a gel filtration column, Superdex 200 Increase 10/300 GL (GE Healthcare), with a mobile phase of 50 mM HEPES buffer (containing 150 mM NaCl, pH 7.5) at a flow rate of 0.75 mL min^−1^.

In the case of TK2268 protein, TK2268 overexpression strain was cultivated in ASW-YT-m1-pyruvate medium at 85°C for 20 h, and cells were collected by centrifugation (5,540 × *g*, 15 min, 4°C). After washing with 0.8× ASW-m1, cells were resuspended in binding buffer (50 mM HEPES buffer, 20 mM imidazole, 500 mM KCl, 10% [vol/vol] glycerol, pH 7.5) and then disrupted by sonication. Insoluble cell debris was removed by centrifugation (12,000 × *g*, 15 min, 4°C). The soluble cell extract was applied to a His GraviTrap column (Cytiva, Marlborough, MA, USA), which had been equilibrated with binding buffer. The TK2268 protein with a His_6_-tag at its C terminus was eluted by elution buffer (50 mM HEPES buffer, 500 mM imidazole, 500 mM KCl, 10% [vol/vol] glycerol, pH 7.5). After concentrating the eluate, it was applied to a gel filtration column, Superdex 200 Increase 10/300 GL (GE Healthcare). The TK2268 protein was eluted with a mobile phase of 50 mM HEPES buffer (pH 7.5) containing 500 mM KCl and 10% (vol/vol) glycerol, at a flow rate of 0.75 mL min^−1^. The purification of TK1094 protein and TK0186 protein followed the method mentioned in a previous paper (15) .

### Enzyme assays

The ability of TK0186p, TK0548p, TK1094p, and TK2268p to catalyze the conversion of OAA to form Asp was analyzed using OAA as an amino acceptor and different amino acids as amino donors. The typical reaction mixture contains 20 µM PLP, 10 mM (sodium) amino acid, 10 mM sodium OAA, 0.6 mM NaCl, and 1 µg mL^−1^ recombinant protein in 50 mM HEPES buffer (pH 7.5). For TK0186p and TK1094p, Ala and Glu were used as amino donors. For TK0548p, aromatic amino acids (Phe, Trp, and Tyr), His and Glu were used as amino donors. In the case of TK2268p, the reaction mixture contains 20 µM PLP, 10 mM sodium Glu, 10 mM sodium OAA, 4.4 mM KCl, 0.045% (vol/vol) glycerol, and 1 µg mL^−1^ recombinant protein in 50 mM HEPES buffer (pH 7.5). The reaction mixture of TK2268p was incubated at 80°C for 0, 1, 2, and 3 min. The reaction mixture of TK0186p, TK0548p, and TK1094p was incubated at 80°C for 0, 15, 30, and 45 min when Phe, Trp, Tyr, His, and Ala were used as amino donors (incubation time was 20, 30, 40, or 50 min when Glu was used as amino donor). The reaction was stopped through cooling the reaction mixture on ice for 10 min. Protein was removed by ultrafiltration using an Amicon Ultra centrifugal filter unit (MWCO 10 K), and the concentration of the generated Asp was measured with HPLC after derivatization. The derivatization mixture (100 µL) contained 10 µL of reaction mixture, 70 µL of solution B (borate sodium hydroxide buffer [400 mM, pH 10.4]), and 20 µL of solution A (8 mg *o*-phthalaldehyde and 10 mg *N*-acetylcysteine were dissolved in 1 mL methanol). After derivatization for 5 min at room temperature, an aliquot (10 µL) of the solution was applied to a COSMOSIL 5C_18_-PAQ column (4.6 mm × 250 mm, 5 µm particle size, Nacalai Tesque) using a Nexera X2 liquid chromatography system with a fluorescence detector RF-20A XS (Shimadzu, Kyoto, Japan). Compounds were eluted with a solution of 20 mM sodium acetate (pH 5.6) and methanol at a flow rate of 0.5 mL min^−1^ (the ratio of sodium acetate to methanol was 7:3). The excitation and emission wavelengths are 350 and 450 nm, respectively.

In kinetic analyses, initial velocities of the aminotransferase reaction were carried out under various concentrations of the substrates. The reaction mixture contained various concentrations of substrates (sodium OAA, sodium 2-oxoglutarate, sodium Glu, and sodium Asp), 20 µM PLP, 4.4 mM KCl, 0.045% (vol/vol) glycerol, and 1 µg mL^−1^ recombinant TK2268p in 50 mM HEPES buffer (pH 7.5). Reactions were carried out at 80°C for 0, 1, 2, and 3 min so that initial velocities could be calculated.

### Asp generation ability in cell-free extract

*T. kodakarensis* KU216, ΔTK0186, ΔTK0548, ΔTK1094, and ΔTK2268 strains were cultivated in ASW-YT-m1-pyruvate medium (each strain was cultivated in three independent bottles) for 20 h and cells were collected by centrifugation (5,540 × *g*, 15 min, 4°C) and washed with 30 mL 0.8× ASW-m1. Cells were disrupted by sonication, and insoluble cell debris was removed (12,000 × *g*, 20 min, 4°C). After exchanging the buffer with 50 mM HEPES (containing 500 mM KCl, 10% [vol/vol] glycerol, pH 7.5) using an Amicon Ultra centrifugal filter unit (MWCO 10 K), the generation of Asp in the cell-free extract was measured. The reaction mixture contained 20 µM PLP, 10 mM sodium Glu, 10 mM sodium OAA, 18.4 mM KCl, 0.37% (vol/vol) glycerol, and 0.018 mg mL^−1^ cell-free extracts in 50 mM HEPES buffer (pH 7.5). The reaction mixture was incubated at 80°C for 0, 1, 2, and 3 min. The reaction was stopped by cooling the reaction mixture on ice for 10 min. Proteins were removed with an Amicon Ultra centrifugal filter unit (MWCO 10 K). The formation of Asp was determined by HPLC after derivatization.

### Growth measurement of host strain and mutant strains

Growth properties of the host strain KU216 and aminotransferase gene disruption strains (ΔTK0186, ΔTK0548, ΔTK1094, and ΔTK2268) were examined in a variety of synthetic media. To understand the contribution of TK0186p, TK0548p, TK1094p, and TK2268p in amino acid catabolism, the host strain and individual disruption strains were cultivated in well-defined synthetic medium ASW-AA-m1-S^0^(Ura^+^). To know whether Asp can be synthesized from other 18 amino acids, cells were cultivated in medium without Asp and Asn (ASW-AA(D^−^N^−^)-m1-S^0^(Ura^+^)). To investigate which compound can restore the growth of cells, the host strain and various mutant strains were cultivated in medium supplemented with Glu, OAA, or both Glu and OAA (ASW-AA(D^−^N^−^)-m1-S^0^(Ura^+^)-Glu, ASW-AA(D^−^N^−^)-m1-S^0^(Ura^+^)-OAA, ASW-AA(D^−^N^−^)-m1-S^0^(Ura^+^)-OAA-Glu). The final concentrations of Glu and sodium OAA were 2 g L^−1^ and 5 g L^−1^, respectively. Cells were precultured in the synthetic ASW-AA-m1-S^0^(Ura^+^) medium for 18 h until the stationary phase and then inoculated into different synthetic media. Cell growth was examined by monitoring OD_660_.

### Tracer-based metabolomic analysis with ^13^C_3_-pyruvate

*T. kodakarensis* KU216 was grown in 5 mL ASW-AA(D^−^N^−^)-m1-S^0^(Ura^+^)-Pyr-Glu supplemented with 0.1% (wt/vol) 1,2,3-^13^C-labeled sodium pyruvate. Cells after 80 h of incubation were collected by centrifugation (20,380 × *g*, 60 min, 4°C) after insoluble elemental sulfur was filtered. Preparation of protein-derived amino acids from the cells and the conditions of subsequent analysis using microfluidic CE-MS are described by Fukuyama et al. (34) . Briefly, protein-derived amino acids were prepared by hydrolyzation with 12N HCl. The purified protein-derived amino acids were analyzed by using a ZipChip CE system (908devices, Boston, MA, USA) coupled with an Orbitrap Fusion Tribrid mass spectrometer (Thermo Fisher Scientific). The obtained MS data were analyzed using Qual Browser in Xcalibur version 4.3.73.11.

## Data Availability

All relevant data are included in the article and supplemental material.

## SUPPLEMENTAL MATERIAL

Supplemental material aem.02017-24-s0001.pdf Table S1; Figures S1 to S4.

ASM does not own the copyrights to Supplemental Material that may be linked to, or accessed through, an article. The authors have granted ASM a non-exclusive, world-wide license to publish the Supplemental Material files. Please contact the corresponding author directly for reuse.
