# Supplementary material for: TK2268 encodes the major aminotransferase involved in the conversion from oxaloacetic acid to aspartic acid in Thermococcus kodakarensis
Source: Appl Environ Microbiol. 2025 Feb 24;91(3):e02017-24. doi: 10.1128/aem.02017-24 (PMC11921379; doi:10.1128/aem.02017-24)
Supplement: Supplemental material — Table S1; Figures S1 to S4. [file aem.02017-24-s0001.pdf]

Supplementary Material

Table S1. Occurrence of TK2268 homologs in Thermococcales species

Thermococcales	Homologs of TK2268
Thermococcus kodakarensis	TK2268
Thermococcus onnurineus	Ton_1259
Thermococcus gammatolerans	TGAM_1838
Thermococcus sibiricus	TSIB_1328
Thermococcus barophilus	TERMP_01282
Thermococcus sp. 4557	GQS_09360
Thermococcus sp. AM4	TAM4_36
Thermococcus cleftensis	CL1_1464
Thermococcus litoralis	OCC_08839
Thermococcus paralvinellae	TES1_1311
Thermococcus nautili	BD01_1614
Thermococcus eurythermalis	TEU_00135
Thermococcus guaymasensis	X802_01935
Thermococcus sp. 2319x1	ADU37_CDS13830
Thermococcus chitonophagus	CHITON_1226
Thermococcus peptonophilus	A0127_04025
Thermococcus piezophilus	A7C91_01730
Thermococcus gorgonarius	A3K92_02075
Thermococcus celer	A3L02_05740
Thermococcus barossii	A3L01_06925
Thermococcus sp. 5-4	CDI07_06895
Thermococcus siculi	A3L11_01055
Thermococcus thioreducens	A3L14_09295
Thermococcus profundus	A3L09_03190
Thermococcus radiotolerans	A3L10_06370
Thermococcus pacificus	A3L08_00190
Thermococcus sp. P6	A3L12_02125
Thermococcus indicus	tFH039_10410
Thermococcus camini	TIRI35C_1690
Thermococcus aciditolerans	FPV09_11205
Thermococcus argininiproducens	K1720_09755
Pyrococcus furiosus DSM 3638	PF0522
Pyrococcus furiosus COM1	PFC_01730
Pyrococcus horikoshii	PH0771
Pyrococcus abyssi	PAB1523
Pyrococcus sp. NA2	PNA2_1412
Pyrococcus yayanosii	PYCH_02880
Pyrococcus sp. ST04	Py04_0704
Pyrococcus kukulkanii	TQ32_03330
Palaeococcus pacificus	PAP_05920

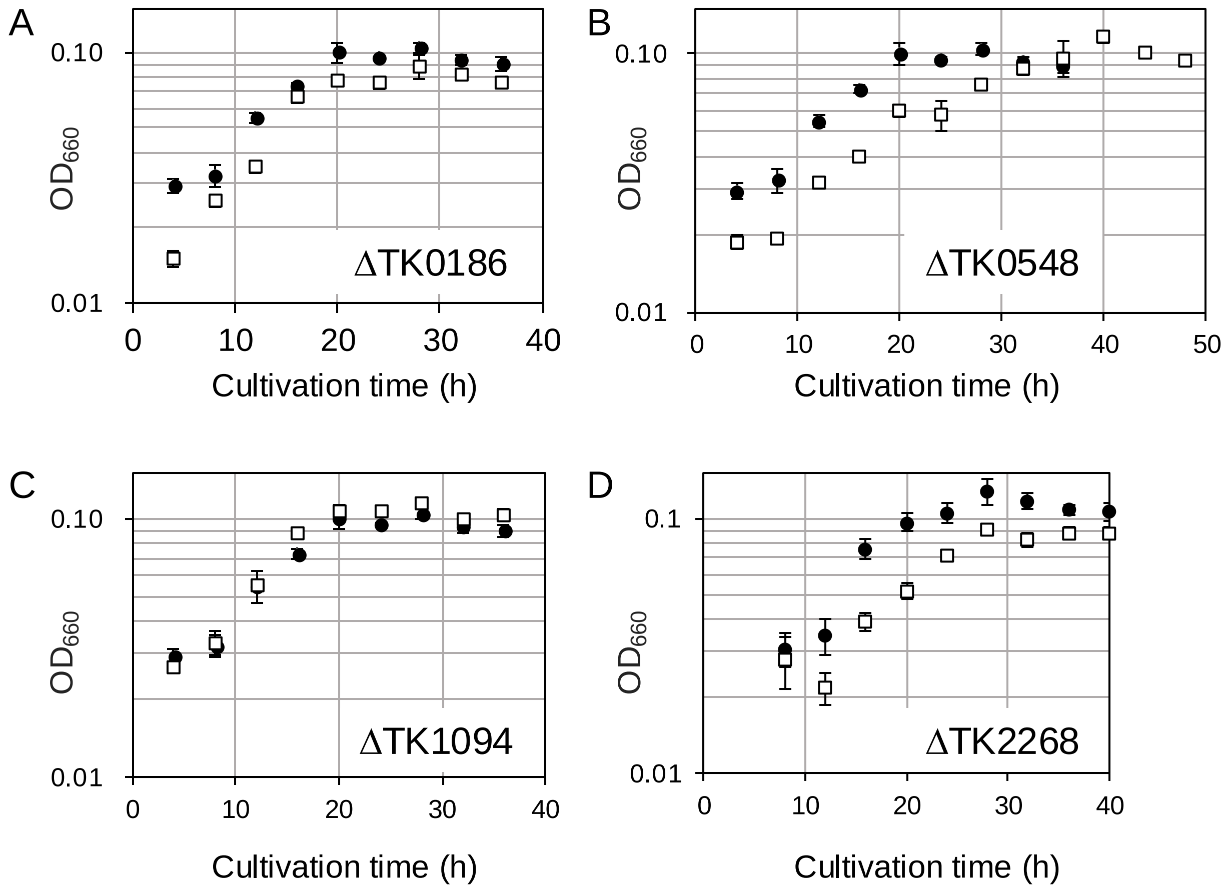

FIG S1. Growth properties of *T. kodakarensis* KU216 and different aminotransferase gene disruption strains in synthetic medium ASW-AA-m1-S^0(Ura^+). Growth of KU216 is indicated with closed circles and those of the disruption strains are indicated with open squares in panels A, ΔTK0186; B, ΔTK0548; C, ΔTK1094; D, ΔTK2268. Error bars indicate the standard deviation values of three biologically independent culture experiments.

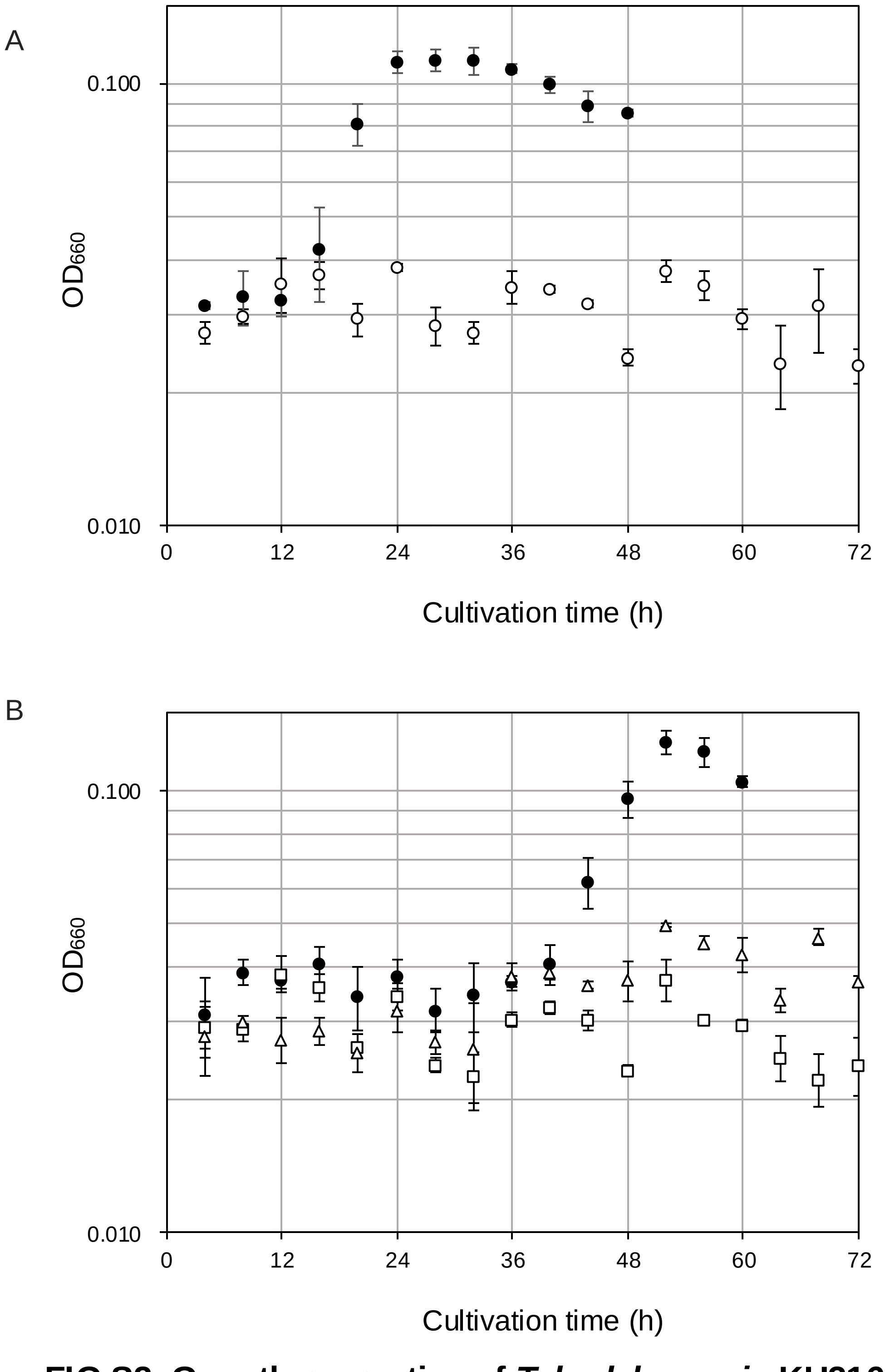

FIG S2. Growth properties of *T. kodakarensis* KU216 in various synthetic media. (A) *T. kodakarensis* KU216 was cultivated in synthetic medium ASW-AA-m1-S^0(Ura^+) (closed circles) and in ASW-AA(D^-N^-)-m1-S^0(Ura^+) (open circles). (B) KU216 was cultivated in synthetic medium ASW-AA(D^-N^-)-m1-S^0(Ura^+) supplemented with Glu alone (open squares), OAA alone (open triangles), or with both Glu and OAA (closed circles). Error bars indicate the standard deviation values of three biologically independent culture experiments.

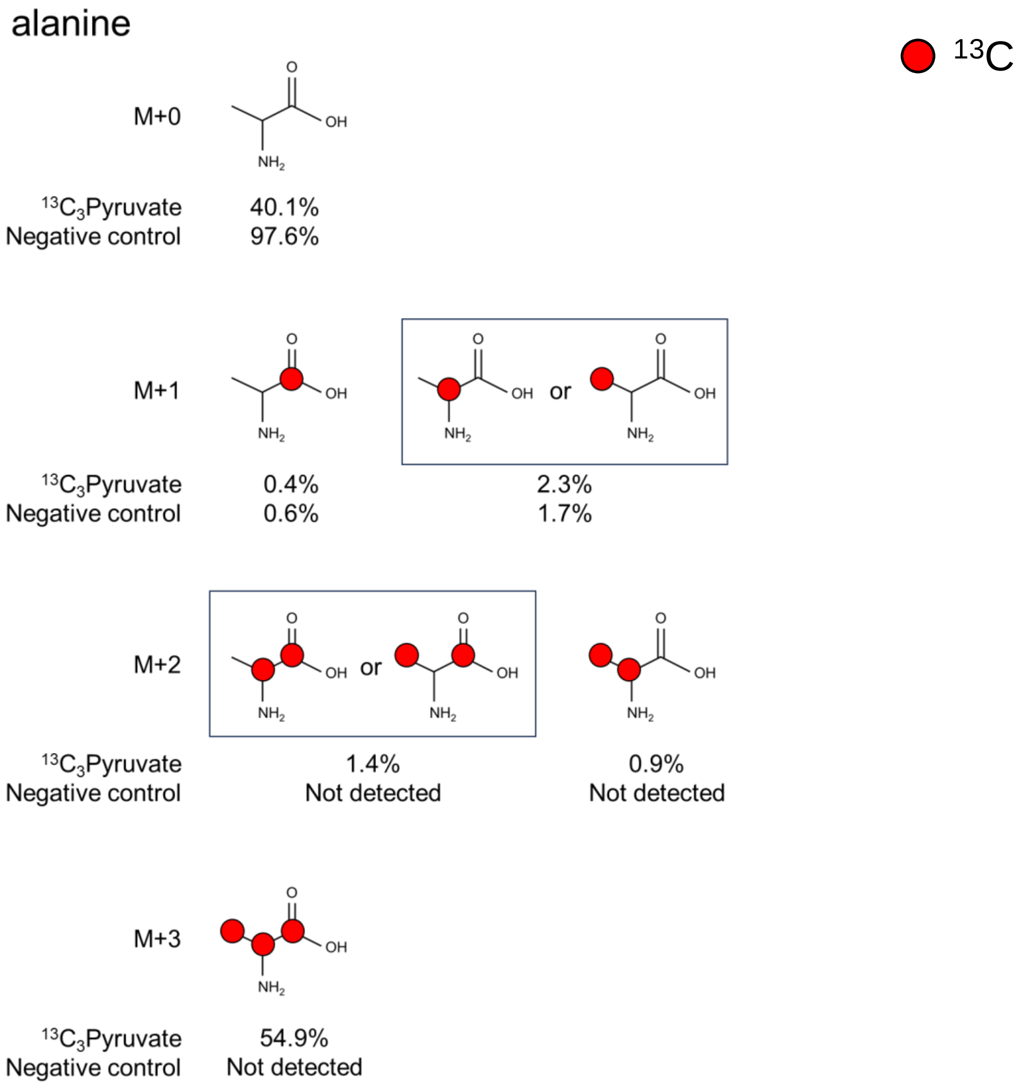

	Isotopomer pattern		Natural abundances of isotopes	*T. kodakaraensis* KU216	
	No. of ^{13}C			$^{13}C_3$ pyruvate Na	Negative control
Amino acid	C-1	Others	Abundance (%)	Abundance (%)	Abundance (%)
$^{13}C_0$ Ala	0	0	96.7	40.1	97.6
$^{13}C_1$ Ala	1	0	1.1	0.4	0.6
	0	1	2.2	2.3	1.7
$^{13}C_2$ Ala	1	1	0.0	1.4	N.D.
	0	2	0.0	0.9	N.D.
$^{13}C_3$ Ala	1	2	0.0	54.9	N.D.

N.D., not detected

FIG S3. The relative abundances of each isotopomer pattern from Ala. The mass fractions for M+0, M+1, M+2, and M+3 represent amino acids containing 0-3 ^{13}C-labeled carbons, respectively. Red circles show ^{13}C-labeled carbon atoms in the carbon skeleton.

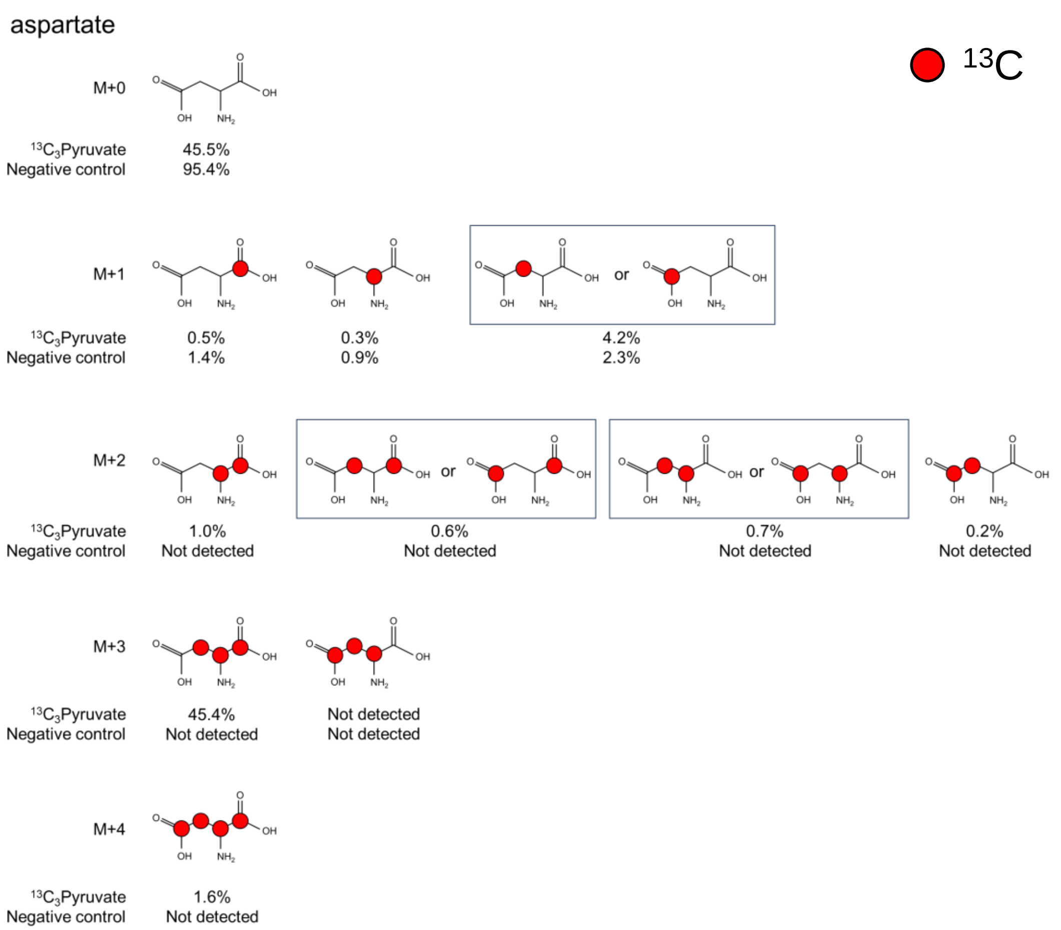

	Isotopomer pattern		Natural abundances of isotopes	*T. kodakaraensis* KU216	
	No. of ^{13}C			$^{13}C_3$ pyruvate Na	Negative control
Amino acid	C-1	Others	abundance (%)	Abundance (%)	Abundance (%)
$^{13}C_0$ Asp	0	0	95.7	45.5	95.4
$^{13}C_1$ Asp	1	0	1.1	0.5	1.4
	0	1	3.2	4.5	3.2
$^{13}C_2$ Asp	1	1	0.0	1.5	N.D.
	0	2	0.0	1.0	N.D.
$^{13}C_3$ Asp	1	2	0.0	45.4	N.D.
	0	3	0.0	N.D.	N.D.
$^{13}C_4$ Asp	1	3	0.0	1.6	N.D.
Amino acid	C-1, 2	C-3, 4	Abundance (%)	Abundance (%)	Abundance (%)
$^{13}C_0$ Asp	0	0	95.7	45.5	95.4
$^{13}C_1$ Asp	1	0	2.1	0.8	2.2
	0	1	2.1	4.2	2.3
$^{13}C_2$ Asp	2	0	0.0	1.0	N.D.
	1	1	0.0	1.3	N.D.
	0	2	0.0	0.2	N.D.
$^{13}C_3$ Asp	2	1	0.0	45.4	N.D.
	1	2	0.0	N.D.	N.D.
$^{13}C_4$ Asp	2	2	0.0	1.6	N.D.

N.D., not detected

FIG S4. The relative abundances of each isotopomer pattern from Asp. The mass fractions for M+0, M+1, M+2, M+3, and M+4 represent amino acids containing 0-4 ^{13}C-labeled carbons, respectively. Red circles show ^{13}C-labeled carbon atoms in the carbon skeleton.
